# The relationship between health literacy and health-related quality of life in Chinese older adults: a cross-sectional study

**DOI:** 10.3389/fpubh.2024.1288906

**Published:** 2024-03-20

**Authors:** Hui Li, Simin Tao, Silu Sun, Ying Xiao, Yongbing Liu

**Affiliations:** ^1^School of Nursing, Chengdu Medical College, Chengdu, Sichuan, China; ^2^School of Nursing, Yangzhou University, Yangzhou, Jiangsu, China

**Keywords:** health literacy, health-related quality of life, association, relationship, older adults

## Abstract

**Background:**

This study aimed to examine the relationship between health literacy and health-related quality of life in older adults.

**Methods:**

A cross-sectional survey design was used. We used a self-administered questionnaire to assess sociodemographic factors of older adults, the Chinese Citizen Health Literacy Questionnaire (HLQC) and the 36-item Chinese version of the Short Form 36 (SF-36) to measure health literacy and quality of life, respectively, among older adults. Between September 2011 and June 2012, information was collected from 1,396 older adults in 44 nursing homes in four cities through face-to-face interviews.

**Results:**

The mean health literacy level of older adults in nursing homes was relatively low (71.74 ± 28.35). Health-related quality of life scores were moderate (104.77 ± 16.92). There were statistically significant differences in the effects of health literacy, education level, former occupation (professional), marital status (widowed) and race on health-related quality of life.

**Conclusion:**

Improving health literacy is considered an important intervention to promote health-related quality of life in older adults in nursing homes.

## Introduction

In recent years, there has been significant growth in older adults population. Globally, the number of people aged 65 and over is expected to increase from 461 million in 2004 to 2 billion in 2050. In China, the number of people aged 65 and over accounts for 13.50 per cent of the total population (1, 2).

With the gradual ageing of the world’s population, the quality of life of older people has received widespread attention, and in recent years, research on health-related quality of life (HRQoL) has become increasingly important. Approximately two-thirds of older people aged >65 years in high-income countries have at least two chronic conditions, 50% have at least three, and 20% have five or more (3–5). Studies have reported that the prevalence of multimorbidity in older people ranges from 55 to 98% (6).

Despite the lack of available data, it is estimated that around 50% of older people in low-and middle-income countries suffer from multiple morbidities (5). In China, people who survive to a relatively late age are more likely to have one or more chronic conditions and to have financial problems accessing the care they need (7, 8). A cross-sectional study in China showed that the prevalence of multimorbidity among older people aged 65 years and over was as high as 35% (9). Multimorbidity is associated with poor quality of life in older people and has been shown in some studies to cause interactions and complications that adversely affect health (5, 10, 11). A new approach to managing and treating ill health in older people is therefore needed (7, 12, 13). Previous research has shown that health literacy is strongly associated with health outcomes in older people. Therefore, assessing the level of health literacy in older people can help health care providers to take targeted action to manage and treat health problems (14).

Health literacy is generally understood as the ability of individuals to maintain and improve their own health by accessing and understanding health information and making appropriate decisions about the use of health-related services. Studies have shown that older people are a particularly vulnerable population in terms of health literacy. Poor physical health (15) and higher mortality (16) are two adverse health outcomes that are significantly associated with low health literacy. It is clear, therefore, that older people are a high-risk, underserved group with inadequate health literacy who require additional attention from healthcare providers.

In medical interventions and health research, HRQoL - which stands for perceived physical health, mental health and functional status - is an important indicator of health status (17, 18). Studies investigating the relationship between health literacy and HRQoL in people with chronic conditions have found no association (19, 20), although other studies have found the opposite. After adjusting for other characteristics, health literacy was found to have a direct effect on many elements of HRQoL (21–23). However, more research is needed to support these findings (24). There is uncertainty about the relationship between health literacy and HRQoL, an important patient-centred outcome that is often assessed in trials (25). We are not aware of other research on HRQoL and health literacy in other populations, such as older people.

If we find evidence of an association between health literacy and HRQoL, health promotion efforts need to take health literacy into account. For example, the finding that HRQoL outcomes in older adults are related to health literacy outcomes raises the question of whether health literacy is modifiable and, if so, whether interventions aimed at improving health literacy will also improve HRQoL in older people.

In a previous study, we examined the relationship between health literacy, health behaviour and health status in Chinese older adults and found that this relationship could be changed by promoting health literacy in older adults (26). The current study aimed to investigate the relationship between HRQoL and health literacy in older people. The significance of these findings is discussed in the context of helping Chinese people to age successfully.

## Methods

### Population research and planning

This cross-sectional study used stratified random cluster sampling to collect data. Four cities - Urumqi, Changji, Karamay and Shihezi - were randomly selected from the Xinjiang province of China, taking into account factors such as population density, geographical size and socio-economic development. All study materials were collected from nursing homes in these four cities. More than 4,500 older adults from 44 nursing homes were included in this study, and the investigator administered questionnaires to 1,452 older adults who met the criteria according to the inclusion and exclusion criteria between September 2011 and June 2012. The investigator administered the questionnaire face-to-face with the older adult, and fully explained the purpose and methodology of the questionnaire to the older adult, explaining the meaning of each item to ensure that they fully understood it. Four postgraduate students from the School of Nursing at the authors’ university carried out the monitoring. All observers underwent a 2-week training period before administering a survey. The entire set of questionnaires was scored and signed by another observer to ensure the integrity of the study.

Age over 60 years (the definition of older age in a developing country), lucidity (participants’ cognitive scores were above the screening cut-offs of the MMSE scale), ability to speak or interact with the researchers, and agreement to cooperate with the researchers after being informed of the purpose of the survey were the inclusion criteria for older adults in the survey. The following were exclusion criteria: refusal to cooperate for their own reasons, psychotic illness, cognitive impairment, serious medical problems (serious physical illness preventing cooperation with the questionnaire), and terminal stage of illness (Table 1).

**Table 1:** Inclusion criteria and exclusion criteria for study subjects.

Inclusion criteria	Exclusion criteria
Age above 60 years (the definition of older age in a developing country)	Refusal to collaborate for one’s reasons;
Clear consciousness (participants’ cognitive scores were above screening cut-offs by MMSE Scale)	Psychotic illnesses
Ability to speak or exchange with researchers	Cognitive impairment
Agreement to cooperate with the inquirers after being informed of the survey’s purpose	Serious medical issues (severe physical illnesses preventing cooperation with the questionnaire)
	Terminal stage of diseases

1,452 older adults, or 32.27% of the total, were randomly selected on the basis of the criteria from 44 care facilities in four locations. The valid response rate for the questionnaires distributed was 96.14%. (1,396 out of 1,452). The sample size, according to Kendall’s method, was 5 to 10 times the research variables. Given that there were 107 independent variables in our study and that 20% of the questionnaires were faulty, the required sample size was 1,070 + 1070* 20% = 1,284 cases. The required sample size of 1,284 was eventually exceeded by a total of 1,396 seniors.

### Examining criteria and a questionnaire

The study was approved by the University Ethics Committee (CMCEC-2022NO.21). The purpose and aims of the study were explained to all participating older people. Each older person was asked to sign a consent form before any information was collected.

The study used a self-administered questionnaire that asked participants about socio-demographic factors, including name, age, gender, race, education level, household income, family size, marital status and occupation.

The Chinese Citizen Health Literacy Questionnaire, created by the China Health Education Centre in 2008, was used to assess health literacy in older persons. The questionnaire has four dimensions: belief, knowledge, conduct, and skill literacy. Each of the 98 items on the questionnaire is worth two points, giving a total score of 196. This survey’s Cronbach’s alpha coefficient, which measures reliability, is 0.904 (27). The level of health literacy in older persons is adequately represented by the Chinese Citizen Health Literacy Questionnaire.

The 36-item Chinese version of the Short Form 36 (SF-36), which is widely used to assess patient health, was used to assess HRQoL (21). Physical function (PF), role limitation due to physical problems (RP), bodily pain (BP), general health (GH), vitality (VT), social function (SF), role limitation due to emotional problems (RE) and mental health were the eight components that made up the SF-36 (MH). In addition, the SF-36 can be divided into two summaries (PCS [PE, RP, BP and GH] and MCS [RE, SF, MH and VT]). HRQoL improves with higher SF-36 scores.

Depending on the cut-off point, the HRQoL score can be categorised into the following three levels: a total score > 117 indicates good HRQoL for older people; a total score of 72–117 indicates fair HRQoL for older adults; and a total score < 72 indicates poor HRQoL for older adults. The Cronbach’s alpha coefficient of this survey is 0.820, indicating good reliability.

### Management and analysis of data

Descriptive statistics were calculated for categorical variables such as health literacy, HRQoL and each dimension score. All data were coded and double-entered by two independent professional data entry staff using EpiData (version 3.1; The EpiData Association, Odense, Denmark). SPSS 18.0 software was used for data analysis (SPSS, Inc., Chicago, IL, United States). Continuous variables that followed a normal distribution were expressed as means and standard deviations. Multiple linear regression analyses were performed to determine the relationship between health literacy and HRQoL. *p* < 0.05 (two-tailed test) was considered statistically significant.

## Results

### Features of a sample

The majority of older people in the final sample (*N* = 1,396) were Han (94.20%) and around half (55.66%) were female. The average age of the participants was 77.37 8.48 years, and the most common level of education was primary school or less (61.53%). 45.85% of the participants had a household income between 2000 and 5,000 RMB, and about half of the families (51.36%) had three to five members. Farmers made up 34.96% of the research participants, and widowed older adults made up 70.56% of the group (Table 2).

**Table 2:** Sociodemographic characteristics in 1396 of older adults.

Sociodemographic characteristics	Percentage (%)
*Age (years)*	
60 **~** 65	102 (7.31)
66 **~** 74	311 (22.28)
75 **~** 84	724 (51.86)
≥ 85	259 (18.55)
*Gender*	
Male	619 (44.34)
Female	777 (55.66)
*Education level*	
Primary school or below	859 (61.53)
Junior high school	220 (15.76)
Senior high school and technical secondary school	193 (13.83)
Graduate and above	124(8.88)
*Household income (Yuan/month)*	
< 500	62(4.44)
500 **~** 1,000	76(5.44)
1,000 **~** 2000	474 (33.95)
2000 **~** 5,000	640 (45.85)
≥ 5,000	144 (10.32)
*Race*	
Han	1,315 (94.20)
Minority	81(5.80)
*Family size (Person)*	
1 **~** 2	266 (19.05)
3 **~** 5	717 (51.36)
≥6	413 (29.58)
*Marital status*	
Unmarried	327 (23.42)
Widowed	985 (70.56)
Divorced	38(2.72)
Married	46(3.30)
*Former Occupation*	
Manager	218 (15.62)
Ordinary staff	121(8.67)
Professionals	237 (16.98)
Service industry employee	101(7.23)
Production staff	231 (16.55)
Farmers	488 (34.96)

### Scores on the four dimensions and health literacy

The four aspect scores (knowledge, beliefs, behaviours and skills) were 32.49 16.88, 22.80 7.41, 9.60 3.91 and 6.22 5.09, respectively. The total health literacy score was 71.74 28.35. All literacy scores other than belief literacy were below the mean (Table 3).

**Table 3:** Health literacy and four dimension scores (*N* = 1,396).

Scale/Subscale	Min	Max	Mean ± SD	95%CI	Possible Range (midpoint) of Scale/Subscale
*Health literacy total scales*	0	149	71.74 ± 28.35	70.25, 73.23	0 to 196 (98)
*Subscales*					
Knowledge literacy	0	78	32.49 ± 16.88	31.61, 33.38	0 to 106 (53)
Belief literacy	0	40	22.80 ± 7.41	22.41, 23.19	0 to 40 (20)
Behavior literacy	0	20	9.60 ± 3.91	9.39, 9.80	0 to 24 (12)
Skills literacy	0	24	6.22 ± 5.09	5.95, 6.48	0 to 26 (13)

### Eight dimension ratings and HRQoL

Total HRQoL scores ranged from 104.77 to 16.92, with the highest score being 143 and the lowest being 55. The physical pain score (75.46 and 21.67) was the highest of the eight dimensions, followed by mental health, vitality, social functioning, role limitations due to emotional problems, role limitations due to physical problems, and physical functioning. General health (54.44 and 18.39) was the lowest. Scale/subscale scores were all above the mean (Table 4).

**Table 4:** HRQOL and eight dimension scores (*N* = 1,396).

Scale/Subscale	Min	Max	Mean ± *SD*	95%CI	Possible Range (midpoint) of Scale/Subscale
*HRQoL total scales*	55	143	104.77 ± 16.92	103.88, 105.66	55–145 (72.5)
*Subscales*					
Physical function (PF)	0	100	54.48 ± 54.48	52.89, 56.06	0–100 (50)
Role limitations due to physical problems (RP)	0	100	55.82 ± 46.59	53.38, 58.27	0–100 (50)
Role limitations due to emotional problems (RE)	0	100	62.92 ± 45.71	60.52, 65.32	0–100 (50)
Social function (SF)	0	100	64.99 ± 20.12	63.93, 66.05	0–100 (50)
Bodily pain (BP)	0	100	75.46 ± 21.67	74.32, 76.59	0–100 (50)
Vitality (VT)	15	100	68.37 ± 16.55	67.50, 69.24	0–100 (50)
Mental health (MH)	12	100	69.77 ± 16.45	68.90, 70.63	0–100 (50)
General health (GH)	0	100	54.44 ± 18.39	53.48, 55.41	0–100 (50)

### Health literacy multivariate analysis links with sociodemographic traits and HRQoL

Multifactor linear regression equations were constructed by including health literacy, education level, occupation, marital status and race. The results showed that the effects of health literacy, education level (compared with primary school and below), occupation (compared with farmers), marital status (compared with married) and race (compared with Han) on QoL were statistically different from each other (Table 5).

**Table 5:** Multiple linear regression analysis of health literacy with sociodemographic characteristics and HRQoL.

Dependent Variable	Independent Variable	Partial regression coefficient	Standard Error	Standardized partial regression coefficient	*t*	*p*
HRQoL	Health literacy	0.096	0.017	0.160	5.606	<0.001
	Education level	1.512	0.488	0.089	3.096	0.002
	Professionals	−2.979	1.210	−0.066	−2.463	0.014
	Widowed	−2.507	0.992	−0.067	−2.527	0.012
	Race	−4.138	1.979	−0.056	−2.091	0.037

Education level (setting dummy variables with reference to “Primary school and below”), Professionals (setting dummy variables with reference to “farmers”), Widowed (setting dummy variables with reference to “married”), Race (setting dummy variables with reference to “Han”).

## Discussion

The current study provides the first account of the relationship between older people’s HRQoL and health literacy from a sample of Chinese nursing homes.

The level of health literacy in the current study (71.74 28.35) was much lower than that reported for older people in the general community (94.88 29.54), which is interesting but not surprising (27). Except for belief literacy, all other dimension scores in the current study were below the mean. Older people in nursing homes had very low health literacy. The age in the current study (77.37 8.48 years) was probably older than the average age in the community (67.23 1.76 years), and the majority of participants (61.53%) had only completed primary school or less. In addition, the educational level of older adults in nursing homes in our study was low. Studies have shown that age and level of education are important determinants of health literacy; those who are older and less educated are less likely to have good health literacy (14, 28, 29). Therefore, health care providers should pay much more attention to the health literacy of older people.

Older people in nursing homes had intermediate HRQoL scores (104.77–16.92) according to the conventional division of HRQoL scores. Of the 1,396 participants, 994 (71.20%) had HRQoL scores between 72 and 117, and 402 (28.80%) had scores below 72. As a result, the majority of older people had moderate HRQoL, suggesting that we should work to further improve HRQoL in older people. Among the eight dimensions, the score for bodily pain was the highest (75.46 21.67) and the score for general health was the lowest (54.44 18.39). As a result, the health status of older people was not ideal. An important indicator of a nation’s economic, social and cultural progress and social civilisation is the HRQoL of older people (30). Therefore, the HRQoL of older adult nursing home residents should be given additional research and attention.

As our results show, the health literacy level of older adults in care homes is not high, and there is a statistical difference in the effect of health literacy on older adults’ HRQoL, from which we can hypothesise that health literacy may be a contributing factor to poorer HRQoL, and that when health literacy levels improve, so does HRQoL. This conclusion provides a basis for considering interventions to develop health literacy, or to reduce the difficulty of reading written materials, and to improve access to healthcare to a relevant level of literacy, using our ‘main effect’ model coefficients. Healthcare professionals can use rapid health literacy screening methods with older community members, as well as in clinical settings, research studies and public health surveillance.

We have discovered a different way to improve the HRQoL of older adults. In China, the ageing problem is getting worse and it has been stated that one of the top agenda items should be how to effectively address population aging. To address population ageing, healthy ageing has emerged as a low-cost, high-efficiency solution. For China, which has a large older adult population and a shortage of medical resources, improving health literacy and promoting healthy ageing is a sensible and constructive course of action.

Our research had a few limitations. Firstly, we only included nursing home residents who were healthy enough to take part in the study. Given that only healthy older people living in care homes were included, the participants may not have been representative. The relationship between health literacy and HRQoL in community-dwelling older people will be further investigated in complementary research. Secondly, health literacy in our study was lower than in previous publications (31); we should broaden the eligibility of the study in the future. This will make it possible to identify potentially significant differences between low and high health literacy groups, and provide new insights into a yet-to-be-discovered relationship between health literacy and HRQoL in the future. Third, this is a cross-sectional study and we have not yet established a causal relationship between health literacy and HRQoL, which will be addressed in future prospective studies. Finally, the study areas selected for this study are all relatively economically and culturally developed cities in the Northern Border Region, so the results obtained may be somewhat biased.

## Conclusion

We have shown the relationship between older people living in care homes and health literacy. The levels of HRQoL and health literacy were also shown. According to the results of the current study, HRQoL in older people is independently influenced by health literacy. We’re still looking at other older people, including community members, patients, and even members of different language and cultural groups.

## Data availability statement

The original contributions presented in the study are included in the article/supplementary material, further inquiries can be directed to the corresponding author.

## Ethics statement

The studies involving humans were approved by Ethics Committee of Chengdu Medical College. The studies were conducted in accordance with the local legislation and institutional requirements. The participants provided their written informed consent to participate in this study.

## Author contributions

HL: Writing – original draft. ST: Data curation, Writing – review & editing. SS: Data curation, Writing – review & editing. YX: Methodology, Writing – review & editing. YL: Writing – original draft, Writing – review & editing.
